# Analysis of the relationship between the gut microbiota enterotypes and colorectal adenoma

**DOI:** 10.3389/fmicb.2023.1097892

**Published:** 2023-04-04

**Authors:** Miwei Lv, Jiawei Zhang, Jiaxin Deng, Jiancong Hu, Qinghua Zhong, Mingli Su, Dezheng Lin, Tian Xu, Xuhao Bai, Juan Li, Xuefeng Guo

**Affiliations:** ^1^Department of Endoscopic Surgery, The Sixth Affiliated Hospital, Sun Yat-sen University, Guangzhou, China; ^2^Guangdong Provincial Key Laboratory of Colorectal and Pelvic Floor Diseases, The Sixth Affiliated Hospital, Sun Yat-sen University, Guangzhou, China; ^3^School of Medicine, Xizang Minzu University, Xianyang, China

**Keywords:** colorectal polyps, gut microbiota, *Prevotella*, enterotype, adenoma

## Abstract

**Introduction:**

The essence of enterotypes is to stratify the entire human gut microbiota, and dysregulation of gut microbiota is closely related to the development of colorectal adenoma. Enterotypes may therefore be a useful target for the prevention of colorectal adenoma. However, the relationship between gut microbiota and colorectal adenoma has not been fully elucidated. In this study, we aimed to analyze the differences in gut microbiome composition between adenoma and control populations.

**Methods:**

We recruited 31 patients with colorectal adenoma and 71 non-adenoma controls. Patient demographics, risk factors, fecal samples from each subject were collected and metagenomic sequencing was performed. LEfSe analysis was used to reveal differences in intestinal microbiome composition. Multiple logistic regression analysis was used to determine the association between enterotypes and colorectal adenoma.

**Results:**

The results showed that *Prevotella* enterotype (enterotype 4) is only present in adenoma group. Logistic regression analysis showed that *Prevotella* enterotype was an independent risk factor for colorectal adenoma.

**Discussion:**

The *Prevotella* enterotype may increase the occurrence of colorectal adenoma through inflammatory association and interference with glucose and lipid metabolism in human body. In conclusion, the differences we observed between different enterotypes add a new potential factor to the development of colorectal adenoma.

## 1. Introduction

Colorectal cancer is one of the third most common types of cancer in the world and it is also the main cause of cancer death (Cao et al., 2021; Sung et al., 2021; Xie et al., 2021). Colorectal cancer is a complex disease influenced by genetics, diet, chronic inflammation, and environmental factors (Dekker et al., 2019; Nguyen et al., 2020; Parmar and Easwaran, 2022). Moreover, advanced polyps are closely related to the occurrence of colorectal cancer (Leslie et al., 2002; He et al., 2018). Therefore, early diagnosis such as screening for polyps is very important to prevent the occurrence and development of colorectal cancer.

Colorectal polyps are caused by the interruption of the normal proliferation and apoptosis cycle of the colon epithelium. Tubular adenomatous polyps and serrated polyps are two common types of precancerous lesions with high malignant potential (He et al., 2020). However, little is known about the composition and role of the microbiome associated with precancerous polyps. Kordahi et al. (2021) revealed the occurrence of colorectal adenoma is closely related to *Bacteroides fragilis*. The gut microbiota is related to human health and can affect human physiological function through immune function and inflammation inhibition, food breakdown and nutrient absorption (Korecka and Arulampalam, 2012; The Human Microbiome Project Consortium, 2012). Pieces of evidence indicated that the dysregulation of human intestinal flora is closely related to the development of a variety of gastrointestinal diseases (Feng et al., 2015; Flemer et al., 2017; Sepich-Poore et al., 2021). However, the gut microbiota varies greatly from individual to individual and the complicative variation limits our understanding of this relationship (Yatsunenko et al., 2012). The presentation of enterotypes is one way to reduce the complexity of these analyzes. Through the analysis of human microbiome genome, Arumugam et al. first introduced the concept of “Enterotypes,” and they found three bacterial groups in human: Bacteroides enterotype, *Prevotella* enterotype, and *Ruminococcus* enterotype (Arumugam et al., 2011). Enterotypes is a classification of the gut microbiota of different populations, indicating that variation in gut microbiota is stratified among individuals. Enterotypes is stable, which is mainly affected by long-term dietary habits. It has no direct relationship with gender, age, geography and cultural background. The enterotypes is characterized by different digestive functions. *Prevotella* enterotype can hydrolyze fiber effectively and has the potential of low fat and low protein fermentation. In contrast, *Bacteroides* enterotype has specific digestive enzymes and is associated with the digestion of animal protein and fat (Christensen et al., 2018). Several associations between enterotypes and disease phenotypes in humans have been reported. Yang et al. (2019) reported that the intestinal microbiome that develops into colorectal cancer in the adenoma-carcinoma sequence can be influenced by its enterotypes. The abnormal biological features of colorectal cancer vary among various intestinal types, especially those dominated by *Escherichia*. The increased abundance of *Bacteroides* is closely related to the occurrence of colorectal cancer, which can be used as an indicator of risk or susceptibility to certain diseases (Zeller et al., 2014; Costea et al., 2018). Based on the various models available, enterotypes could in some cases provide some important indications. The integration of enterotypes into various models can be a tool to better understand the presence of an individual’s susceptibility to certain diseases (Di Pierro, 2021).

For the risk factors of adenoma development, most studies are still carried out on specific microflora (Kordahi et al., 2021). Due to the complex diversity of intestinal microflora, it is difficult to coordinate the differences among different individuals through one or several types of bacteria. In contrast, enterotypes is an emerging classification method to express the characteristics of human intestinal flora. However, few studies have directly investigated the association between enterotypes and adenoma. Therefore, we aim to systematically study the microbial composition of human fecal samples at various points based on enterotypes. Taxonomic and enterotypes data were used to investigate whether there was a difference in enterotypes between intestinal adenoma and control patients, and whether there was an association between enterotypes and intestinal adenoma.

## 2. Materials and methods

### 2.1. Ethical statement

Informed consent of all participants was obtained for this study. The collection of stool samples and data analysis were approved by the institutional ethics board of the Sixth Affiliated Hospital, Sun Yat-sen University (NO. 2021ZSLYEC-290). The study was also conducted in accordance with the Declaration of Helsinki (revised in Fortaleza, Brazil, October 2013).

### 2.2. Subjects

All participants will be recruited in the Sixth Affiliated Hospital of Sun Yat-Sen University from September 2021 to February 2022. In this population-based study, 102 human fecal samples were prospectively collected at the Sixth Affiliated Hospital of Sun Yat-Sen University. The subjects were a high risk population of colorectal cancer aged 40–80 years who lived in coastal areas of Guangzhou City. The exclusion criteria for our study were as follows: (1) History of gastrointestinal surgery. (2) Functional or metabolic bowel lesions within the past 3 months. (3) Had taken medication for infectious diseases in the past 1 month. (4) Had undergone gastroenteroscopy within the past 6 months. (5) Had taken probiotics in the past 1 month. (6) A history of familial adenomatous polyposis and inflammatory bowel disease. A questionnaire survey was conducted on all the subjects using a Case Report Form, including age, gender, history of surgery, height, weight, eating habits, previous medical history, medication history, and consumptive history of tobacco and alcohol. All subjects in this study received routine bowel preparation, including polyethylene glycol electrolyte lavage powder. Enteroscope was performed by 6 experienced endoscopists.

### 2.3. Fecal sample collection

Stool samples were kept at 4°C immediately after defecation and transported to the laboratory within 12 h of defecation and stored at −80°C until analysis. After stool collection, colonoscopy was performed for patients eligible for inclusion, and polyps were pathologically classified. Fecal samples, whether transported short or long distances, must be kept in a container filled with liquid nitrogen and supervised by a person.

### 2.4. Gut microbiota

Absorb 1,000 μl CTAB lysate into 2.0 ml EP tube and add it to lysozyme. Then the appropriate amount of samples were added to the lysate in a 65°C water bath. Reverse mix several times during this period to make the sample full Cracking. Supernatant was centrifuged, phenol (Ph8.0): chloroform: isoamyl alcohol (25:24:1) was added, mixed upside down, and centrifuged at 12000 rpm for 10 min. Absorb supernatant into 1.5 ml centrifuge tube and add isopropyl alcohol. Rocking up and down, settling at −20 degrees. Centrifuge at 12000 rpm for 10 min and pour out the liquid. Wash with 1 ml 75% ethanol twice, the remaining small amount of liquid can be centrifuged again and collected, and then sucked out with the tip of the gun. Add ddH2O to dissolve DNA samples and incubate at 55–60°C for 10 min to aid dissolution if necessary. RNase A 1 μl digested RNA was added and placed at 37°C for 15 min. DNA purity and integrity were analyzed by agarose gel electrophoresis. After qualified DNA samples were detected, the Covaris (Covaris S2 System, Massachusetts, United States) ultrasonic crusher was used to randomly interrupt the DNA samples, and then the whole library preparation was completed by terminal repair, adding A tail, adding sequencing joint, purification, PCR amplification and other steps. DNA concentrations were measured with a Qubit 2.0 Fluorometer (Life Technologies, Carlsbad, CA, United States). After qualified library detection, different libraries are pooled to flowcell according to the requirements of effective concentration and target off-machine data volume. After cBOT clustering, Illumina (Illumina, San Diego, CA, United States) PE150 (2×150) high-throughput sequencing platform is used for sequencing.

### 2.5. Metagenomic sequencing

Illumina Novaseq platform was used for double-ended sequencing of sequencing samples. The original sequencing data were preprocessed, including quality control (Trimmomatic parameter: ILLUMINACLIP: adapters_path: 2:30:10, SLIDINGWINDOW: 4:20, MINLEN: 50) and dehoing sequence (Bowtie2 parameter: very sensitive) to obtain effective sequences (clean data) for subsequent analysis. Main steps of the analysis process: (1) Data quality control and dehosting sequence: KneadData software was used for quality control of original data (based on Trimmomatic) and dehosting (based on Bowtie2). Before and after KneadData, FastQC was used to test the rationality and effect of quality control. (2) Species notes: Kraken2 and self-built microbial nucleic acid database (screening the sequences belonging to bacteria, fungi, archaea and viruses in NCBI NT nucleic acid database and RefSeq whole genome database) were used to annotate samples, and then Bracken was used to estimate the actual abundance of species in the sample. (3) Cluster analysis of abundance based on species abundance table: PCoA and NMDS dimension reduction analysis (species only), sample cluster analysis; When grouping information was available, LEfSe biomarker excavation analysis and comparative analysis of metabolic pathways were performed to detect differences in species composition and functional composition among samples.

### 2.6. Enterotypes of analysis

Enterotypes were identified by plotting the log-transformed abundance of Bacteroides versus the log-transformed abundance of *Prevotella*, which were calculated using the diptest package in R4.05 (The R4.05 Project for Statistical Computing, Vienna, Austria). Samples were clustered using Jensen-Shannon distance and partitioning around medoid (PAM) clustering (Liang et al., 2017). Optimal number of clusters was estimated using CalinskiHarabasz (CH) index (Arumugam et al., 2011). We used the silhouette validation technique for assessing the robustness of the clusters. The clustering quality of PAM was assessed in silhouette. Linear discriminant analysis (LDA) effect size (LEfSe, v1.0) was used to analyze the significant differences in relative abundance of gut microbiota categories related to the patients with the enterotype-1 group and the controls with the enterotype-2 group. Linear discriminant analysis (LDA) effect size (LEfSe) was used for the identification of the different markers, and the LDA threshold was set to be >4 (Segata et al., 2011). Methods Nonparametric test and linear discriminant analysis were combined to find biomarkers of each group. LEfSe searched for the biomarker function of each group (LDA > threshold function, with higher abundance in the corresponding group and lower abundance in other groups). That is, functions that are significantly more abundant in this group than in the other groups.

### 2.7. Statistical analysis

The continuous variables with normal distribution were expressed as the mean ± standard deviation (SD), and the variables with nonnormal distribution were presented as the median (interquartile range). The categorical variables were presented as numbers (%). The normal distribution of the data was tested using the Kolmogorov–Smirnov test. Continuous, ordinal and categorical variables are expressed as mean ± standard deviation, median and interquartile range, and frequency or proportion (percentage), and were compared using the unpaired Student *t*-test, Wilcoxon rank-sum test and *χ*^2^ test, respectively. Variables that had a value of *p* <0.05 in univariate analysis were subjected to multivariate logistic regression analysis. Multiple logistic regression analysis was used to determine the association between enterotypes and colorectal adenoma. The area under the ROC curve (AUC) with 95% confidence interval, sensitivity, and specificity were calculated. All *p*-values are two-sided and *p* < 0.05 was considered statistically significant.

## 3. Results

### 3.1. Demographic characteristics

According to the results of colonoscopy, all subjects were divided into adenoma group and control group. A total of 31 patients with colorectal adenoma and 71 controls were included. In the adenoma group of 31 cases, there are 31 cases of tubular adenomas. Studies have shown that the size, number, villous structure, and grade of dysplasia of colorectal adenomas are closely associated with a higher frequency of colorectal cancer development (Heitman et al., 2009; Wieszczy et al., 2020). In our study, all of the adenoma patients were tubular adenomas with no adenomas found in the basal incisal margin. Besides, 23 patients had single tubular adenomas and 8 patients had multiple tubular adenomas. Table 1 was created to describe radenomas size data in detail. The male/female ratio of the adenoma and control groups was 17/14 and 26/45, respectively. In order to exclude the influence of confounding factors on the results, multivariate logistic regression analysis was performed for variables with *p* value <0.05 in univariate analysis. Table 2 was created to describe relative data in detail. The results of multivariate regression analysis showed that there were no statistical differences between the two groups in terms of gender, age, BMI, eating habits, history of diabetes, and smoking status(*p* > 0.05). The mean age of the adenoma group was 50.57 ± 6.841 years and that of control group was 52.46 ± 8.261 years.

**Table 1:** Tubular adenoma size in adenoma patients in the adenoma group.

Adenoma size (cm)	Quantity
0.1	3
0.2	6
0.3	5
0.4	10
0.5	2
0.6	3
1.0	1
1.2	1

**Table 2:** Demographics and baseline characteristics of patients between control group and adenoma group.

	Control group (*n* = 71)	Adenoma group (*n* = 31)	*p* value	Multivariate		
				Odds ratio	95% confidence interval	*p* value
Age (year, mean ± SD)	52.46 ± 8.261	50.57 ± 6.841	0.271			
BMI, kg/m^2^ (mean ± SD)	22.53 ± 3.00	23.45 ± 2.93	0.441			
Gender, *N*%			0.087			
Male	26 (36.6)	17 (54.8)				
Female	45 (63.4)	14 (45.2)				
Diabetes, *N*%			0.427			
Yes	4 (5.6)	0 (0.0)				
No	67 (94.4)	31 (100.0)				
Hypertension, *N*%			1.000			
Yes	8 (11.3)	3 (9.7)				
No	63 (88.7)	28 (90.3)				
Smoking, *N*%			1.000			
Yes	8 (11.3)	3 (9.7)				
No	63 (88.7)	28 (90.3)				
Drinking, *N*%			**0.036**	8.786	1.811–42.622	**0.007**
Yes	3 (4.2)	6 (19.4)				
No	68 (95.8)	25 (80.6)				
Diet, *N*%			0.535			
Balanced	17 (23.9)	21 (20.3)				
Unbalanced	54 (76.1)	5 (79.7)				

### 3.2. Cluster numbers and characteristics of enterotypes

**Figure 1:**
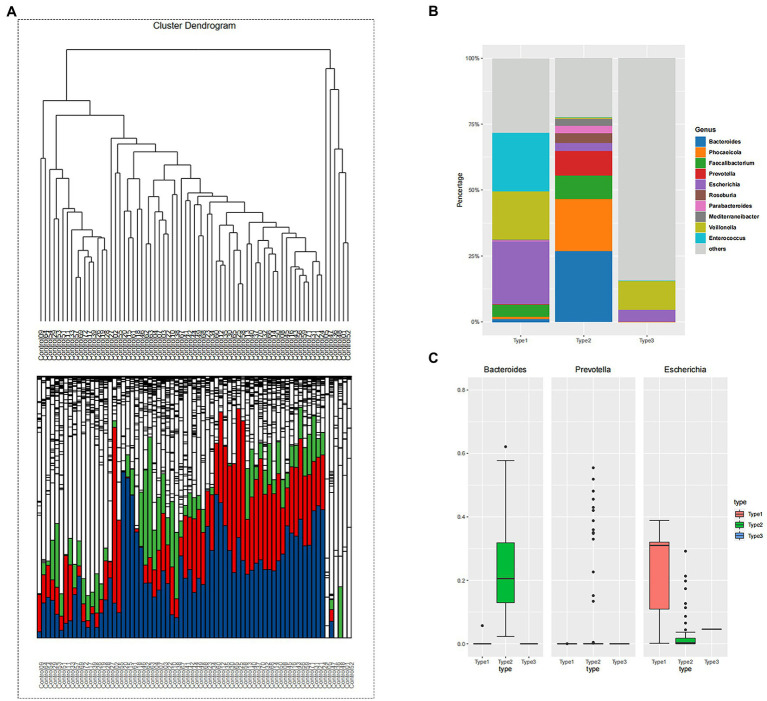
**(A)** The hierarchical clustering result of control group. The clustering results reflect the distance between the samples, allowing the samples to be divided into three distinct enterotypes and showing the relative abundance of the bacteria contained in the sample at the level of the genus contained in each branch. Enterotype-*Escherichia*: 19; Enterotype-*Bacteroides*: 46; Enterotype-*Veroniella*: 6. (blue: *Bacteroides*; green: *Faecalibacterium*; red: *Prevotella*). **(B)** The samples were divided into three groups according to the clustering results of control group, and the top 10 genera contained in each group were displayed. Bacterial community of three enterotypes: a bacterial proportion in the three enterotypes. **(C)** The grouping box diagram shows the percentage content of the three bacterial genera in the three groups clustered by samples in control group.

β-diversity matrices were used to identify the enterotypes in fecal samples *via* clustering methods: partitioning around medoids (PAM). In order to determine the difference in enterotypes between the two groups, hierarchical cluster analysis was performed for the adenoma group and control group, respectively. The unsupervised classification method produced a dendrogram of the clustering results of control group (Figure 1A). Stratified cluster analysis showed that the control group was divided into three enterotypes, classified as containing *Escherichia* enterotype (enterotype 1), *Bacteroides* enterotype (enterotype 2), and *Veroniella* enterotype (enterotype 3). There were 19 cases of *Escherichia* enterotype, 46 cases of Bacteroides enterotype and 6 cases of *Veillonella* enterotype in control group (Figure 1A). The bar charts (Figure 1B) illustrate the relative abundance of bacteria among three enterotypes. Enterotype 1 mainly contains three dominant genera: *Escherichia* (24%), *Enterococcus* (22%) and *Veillonella* (18%); Enterotype 2 mainly contains *Bacteroides* (27%), *Phocaeicola* (20%) and *Prevotella* (9%); and Enterotype 3 mainly consists of two dominant genera: *Veillonella* (11%) and *Escherichia* (5%) (Figure 1B). Our study found that a new enterotypes, *Veillonella* enterotype, emerged in the control group as opposed to the traditional enterotypes. Figure 1C showed the predominance of the three most abundant bacteria in the three enterotypes of control group. The abundance of *Escherichia* in enterotype 1 was higher than that of enterotype 2 and 3, and the abundance of *Bacteroides* in enterotype 2 was higher than that of enterotype 1 and 3. These results correspond to the bacterial abundance in enterotypes 1 and 2.

**Figure 2:**
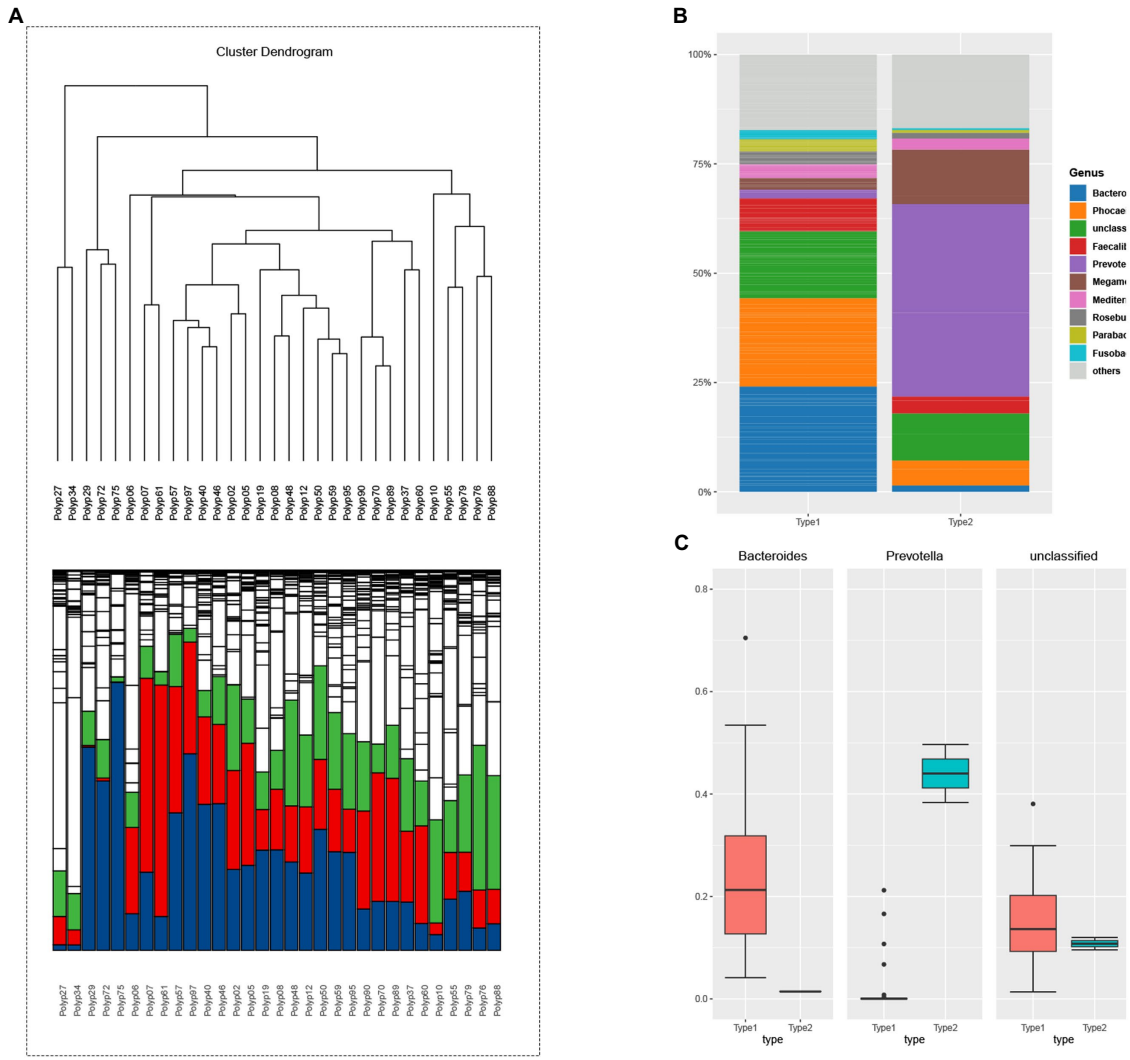
**(A)** The hierarchical clustering result of adenoma group. The clustering results reflect the distance between the samples, allowing the samples to be divided into two distinct enterotypes and showing the relative abundance of the bacteria contained in the sample at the level of the genus contained in each branch. Enterotype-*Bacteroides*: 10; Enterotype-*Prevotella*: 49. (blue: *Bacteroides*; green: *Prevotella*, red: *Faecalibacterium*). **(B)** The samples were divided into two groups according to the clustering results of adenoma group, and the top 10 genera contained in each group were displayed. Bacterial community of two enterotypes: a bacterial proportion in the two enterotypes. **(C)** The grouping box diagram shows the percentage content of the three bacterial genera in the three groups clustered by samples in adenoma group.

The unsupervised classification method produced a dendrogram of the clustering results of adenoma group (Figure 2A). Stratified cluster analysis showed that the adenoma group was divided into two enterotypes. Therefore, then two enterotypes were classified as Bacteroides enterotype (enterotype 2) and *Prevotella* enterotype (enterotype 4). There were 5 cases of *Bacteroides* enterotype and 26 cases of *Prevotella* enterotype (Figure 2A). The dominant bacteria were enterotype 2: *Bacteroides* (24%), *Phocaeicola* (20%) and Unclassified (15%); enterotype 4: *Prevotella* (44%), *Megamonas* (12%) and Unclassified (11%) (Figure 2B). Figure 2C shows the predominance of the three most abundant bacteria in the two enterotypes of adenoma group. *Prevotella* enterotype only exists in the adenoma group.

### 3.3. Microbiota differences between the adenoma group and the control group of Bacteroides enterotype

**Figure 3:**
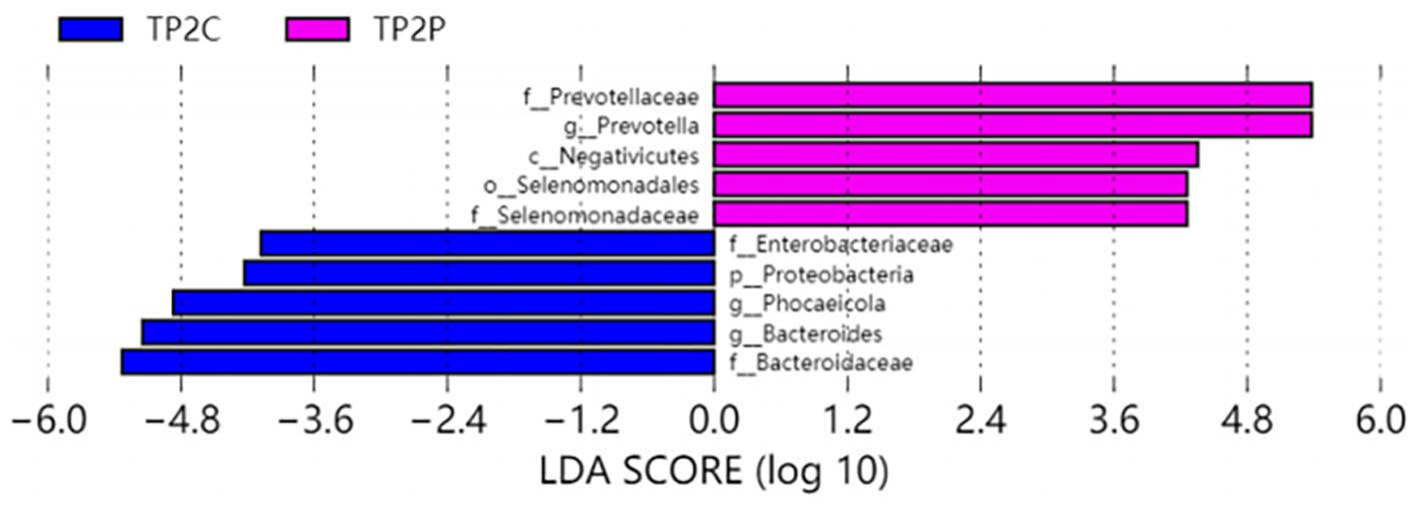
Differently abundant taxa identified using LEfSe analysis. Visualization of only taxa meeting an LDA threshold >4.

**Figure 4:**
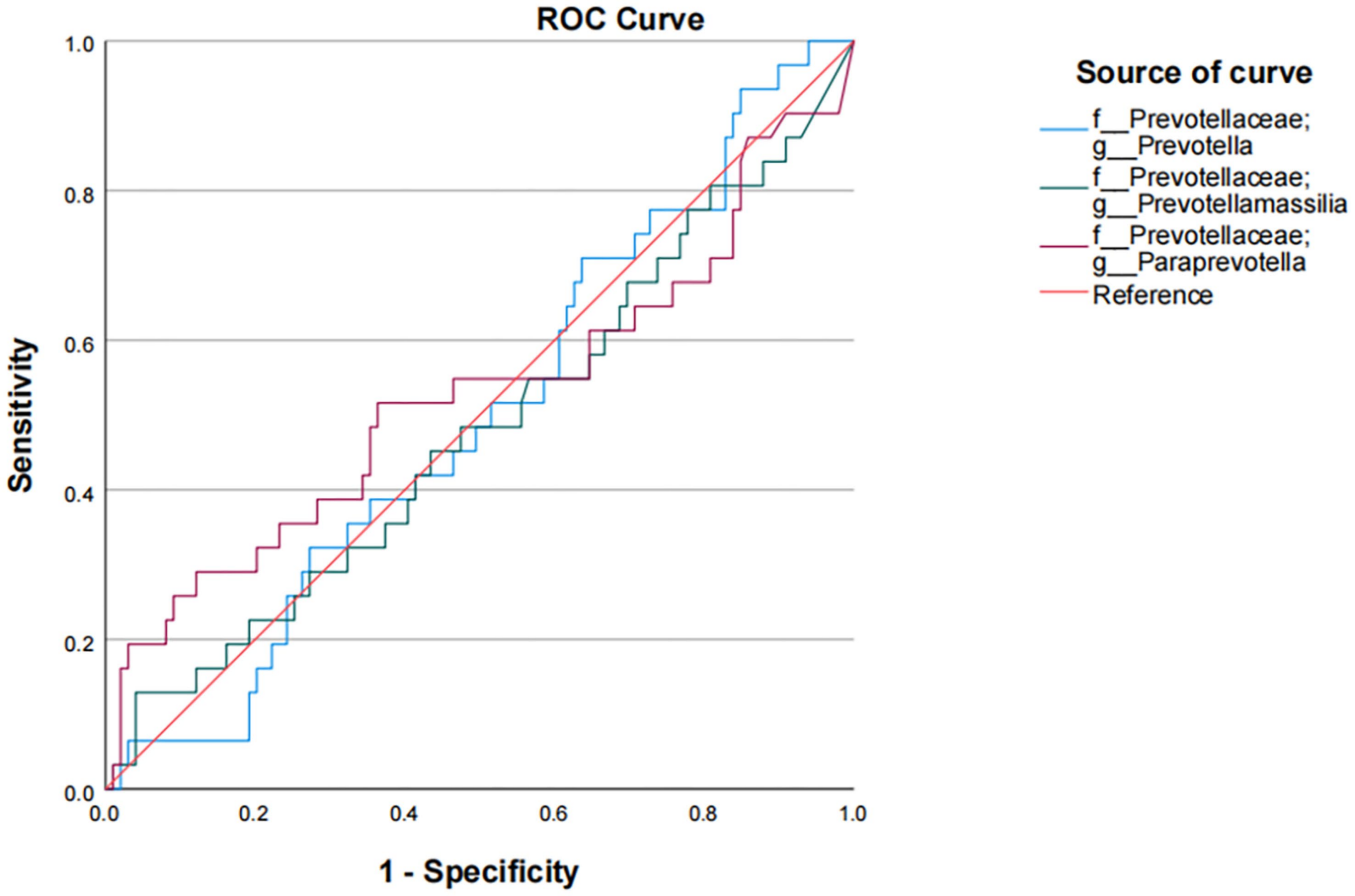
Logistic regression analysis of the association of f_Prevotellaceae and g_Prevotella with colorectal adenoma.

In our study, *Prevotella* enterotype was only present in the adenoma group. Therefore, we believe that *Prevotella*, as the dominant bacteria of *Prevotella* enterotype, is also a characteristic bacteria in adenoma group. *Bacteroides* enterotype in the adenoma group and the control group were analyzed by LEfSe to find different bacteria and the control group in Bacteroides enterotype was named TP2C, and the adenoma group was named TP2P. Through analysis, the flora difference between control group in Bacteroides enterotype (TP2C) and adenoma group in Bacteroides enterotype (TP2P), the abundance of f_*Prevotellaceae* and g_*Prevotella* in the adenoma group was significantly higher than that in the control group (Figure 3). And g_*Prevotella* happens to be the dominant bacterium in *Prevotella* enterotype. Therefore, to further explore the predictive ability of characteristic bacteria to diseases, we used f_*Prevotellaceae* detected in the samples; g_*Prevotella*, f_*Prevotellaceae*; g_*Prevotellamassilia* and f_*Prevotellaceae*; g_*Paraprevotella* and use ROC prediction curve to predict them. The results showed that adenoma, the prediction ability was weaker, and regression logic analysis showed that AUC = 0.498, AUC = 0.480, AUC = 0.527 (Figure 4). Therefore, we believe that the occurrence of adenomas is the result of the influence of multiple factors, and enterotypes is better in predicting adenomas.

### 3.4. Logistic regression analysis of the association of *Prevotella* enterotype with colorectal adenoma

**Figure 5:**
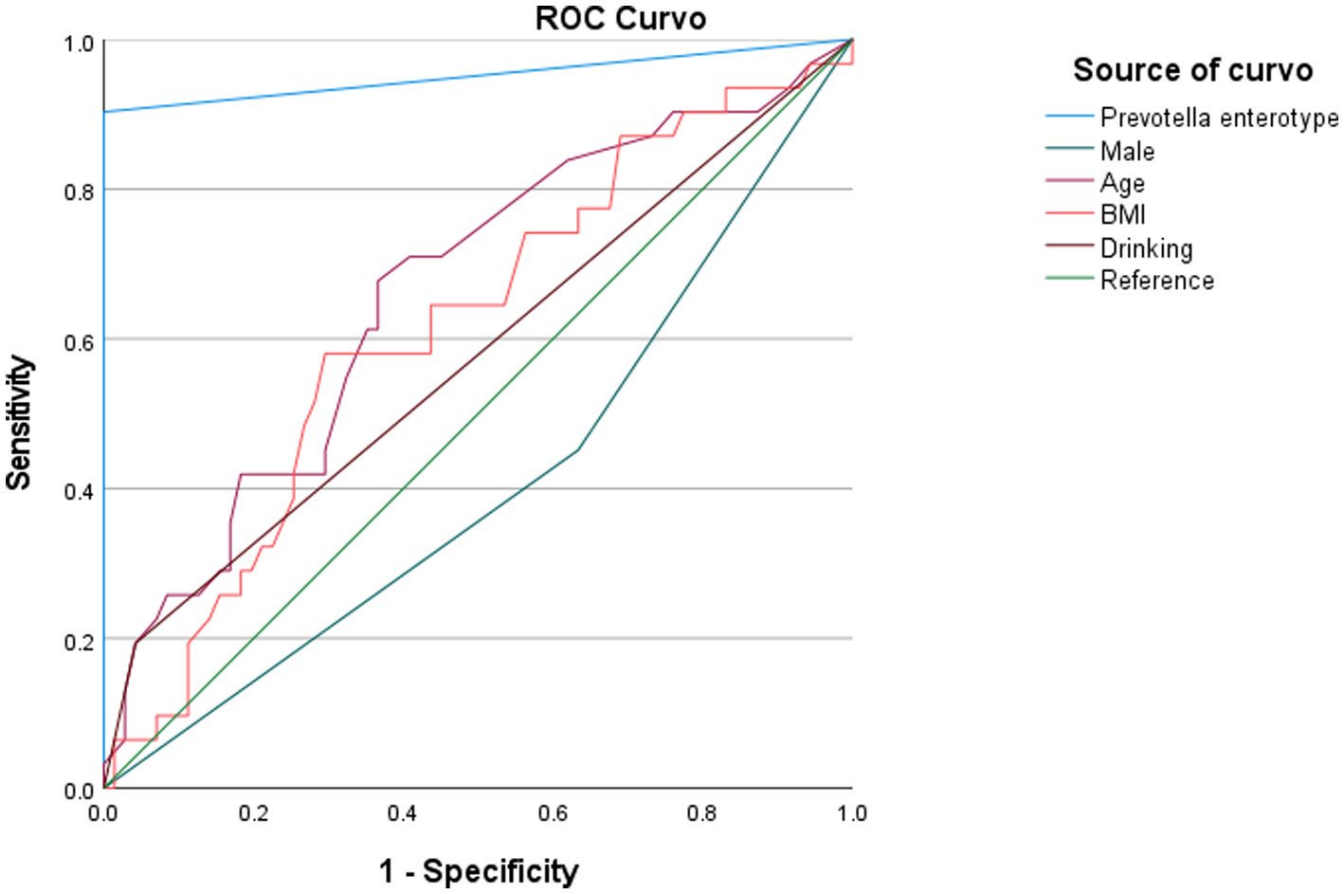
Diagnostic potential of *Prevotella* enterotype in predicting the incidence of colorectal adenoma. AUC, area under curve; BMI, body mass index.

In this study, we developed three different models to observe the relationship between enterotypes and adenomas (Table 3). In model 1, we first simply addressed the role of *Prevotella* enterotype in colorectal adenoma and found that *Prevotella* enterotype presented a significantly increased odds ratio (OR) for colorectal adenoma (OR = 23.856, *p* = 0.001). Then, we adjusted the gender, age and BMI of Model 1 to produce Model 2. In model 2, age, sex and BMI were not associated with colorectal adenoma. To exclude the effects of other factors on the results, we further adjusted model 2 for drinking (model 3). Finally, it was observed that *Prevotella* enterotype still increased the odds ratio of increased (OR) for colorectal adenoma (OR = 23.970, *p* = 0.023). Stepwise multivariable logistic regression analyzes (model 1, model 2 and model 3) showed that *Prevotella* enterotype is independently associated with the presence of colorectal adenoma (Table 3). Properly fit the statistical model 3 and *Prevotella* enterotype as multivariable, predict colorectal adenoma by logical regression, and use ROC curve to evaluate. As shown in Figure 5, *Prevotella* enterotype can predict the risk of colorectal adenoma (AUC = 0.952; *p* < 0.001; Figure 5).

**Table 3:** Logistic regression analysis of the association of *Prevotella* enterotype with colorectal adenoma.

Characteristics	Model 1 OR (95% CI)	*p* value	Model 2 OR (95% CI)	*p* value	Model 3 OR (95% CI)	*p* value
*Prevotella* enterotype	23.856 (23.120–24.907)	0.001	23.997 (23.330–44.326)	0.018	23.970 (23.341–51.623)	0.023
Male			−0.762 (−20.382–17.159)	0.304	−0.515 (−19.784–18.236)	0.435
Age (years)			−0.005 (−0.143–0.120)	0.920	−0.005 (−0.187–0.149)	0.927
BMI (kg/m^2^)			0.158 (0.035–0.681)	0.100	0.146 (−0.110–0.701)	0.121
Drinking					1.135 (−18.270–21.856)	0.176

## 4. Discussion

Our study showed that the *Prevotella* enterotype exists in patients with colorectal adenoma, and is considered to be a characteristic enterotype in colorectal adenoma. Most colorectal cancer patients develop through colorectal polyps. Colorectal polyps can develop into highly dysplasia through sufficient mutations and eventually invade the submucosa (Shaukat et al., 2020). Intestinal flora has been reported to play an important role in promoting the development of colorectal adenomatous polyps to colorectal cancer (Tjalsma et al., 2012). It has been reported that a significant increase in *Fusobacterium_mortiferum* is closely related to the development of colorectal polyps (Liang et al., 2020). The relationship between intestinal flora and colorectal polyps is limited due to the great variation of intestinal flora among individuals.

Methods involving metagenomic sequencing in our study. Compared to assembly-based species annotation, metagenomic species annotation methods may be more comprehensive and accurate for reads-based methods. However, metagenomic analysis also has a disadvantage, It is based on existing databases and cannot detect new genes in samples, so assembly-based analysis and read-based analysis each have advantages and disadvantages.

Enterotypes as a new concept to characterize human intestinal flora, has been widely used in various research in recent years. Arumugam et al. (2011) proposed that human intestinal microbial community can be divided into three different types “enterotypes”. The enterotypes of an individual can be highly variable, and the concept of enterotypes has important implications for how disease studies related to the microbiome are conducted (Knights et al., 2014). At present, more and more studies show that intestinal pattern is related to the occurrence of various diseases. Studies have shown that *Bacteroides* enterotype may be associated with the development of anxiety and depression syndrome (Valles-Colomer et al., 2019). Sobhani et al. (2011) found that the significant elevation of *Bacteroides*/*Prevotella* populations in colorectal cancer patients seemed to be related to the elevation of IL17-producing cells in the mucosa of cancer patients. In addition, studies have indicated that *Bacteroides* can affect the metabolism of nutrients in the body through dietary habits in patients with inflammatory bowel disease (Weng et al., 2019). However, few studies have been conducted on the correlation between enterotypes and colorectal adenoma. In our study, the incidence of colorectal adenomas was independent of age by univariate baseline characteristics analysis (*p* = 0.271). But in previous studies, it is considered that the occurrence of adenoma is correlated with the age (Wong et al., 2020). However, when Arumugam et al. first proposed enterotypes, they proposed that enterotypes are independent of ethnicity, sex, age, and BMI, but are driven by populations together and dominated by dominant bacteria. After the overall classification of the samples, the samples were first divided into two groups, but this rough classification obviously lacked the characteristics to judge the subsequent adenoma. Therefore, after the enterotypes analysis of the control group and the adenoma group, we found that the control group could be divided into three enterotypes: *Escherichia* enterotype, Bacteroides enterotype and *Veroniella* enterotype. The adenoma group was divided into Bacteroides enterotype and *Prevotella* enterotype. In the previous study, Arumugam et al. (2011) proposed three dominant bacteria for enterotypes: Bacteroides enterotype, *Prevotella* enterotype and *Ruminococcus* enterotype. As for the possible classification of other enterotypes, Liang et al. (2017) found that *Enterobacteriaceae* could be a new subtype of enterotypes in the Asian population. In a study based on a Chinese population, Lu et al. (2021) identified four enterotypes in 2678 healthy Chinese people, three of which were enriched in *Prevotella*, Bacteroides and Escherichia coli, while the fourth was a mixed type with no dominant genus. In this study, the three enterotypes were consistent with those seen in previous studies. Our results showed that the Bacteroides enterotypes included adenoma patients and control people, the *Prevotella* enterotypes was only present in the adenoma group, and the *Escherichia* enterotypes type was only present in control people. The differences in the distribution and formation of enterotypes in different regions may be related to geographical environment, altitude, local urbanization process, diet and other factors (Lu et al., 2021). This also indicates that the research on enterotypes may be different with different sample ranges, and people in each region may have enterotypes distribution with corresponding regional characteristics.

In this study, we also identified a new enterotypes in the control group, the *Veillonella* enterotype. Because we only found it in control people, we wanted to know if *Veillonella* enterotype had an inhibitory effect on adenoma, or if it had a protective effect on the human gut. A study based in Japan found that *Veillonella* is commonly found and studied as a human oral colonizer (Mashima et al., 2021). *Veillonella* is commonly found in the natural cavities of animals and humans, and is found in the mouth, pharynx, respiratory tract and digestive tract. Its ability to adhere to surfaces or interact with other bacteria and form biofilms is critical to the composition and function of the gut and oral microbiome, particularly in the oropharynx and gut (Mashima et al., 2021). In addition, it has also been suggested that *Veillonella* may play a protective and beneficial role in early childhood immune system development (Hasegawa et al., 2016). *Veillonella* can be metabolized in the human body by using short-chain organic acids as a carbon source and energy source (van den Bogert et al., 2013). The interaction between *Veillonella* and the host has been implicated in the pathogenesis of gastrointestinal diseases and chronic inflammation. The presence of typical oral microorganisms (including *Veillonella*) in the intestinal mucosa has been associated with a variety of pathologies, including colorectal cancer and inflammatory bowel disease (Geng et al., 2014; Flemer et al., 2018). Nitrate is a signature metabolite of inflammation, and *Veillonella* has respiratory nitrate reductase, which is capable of anaerobic respiration. Nitrate respiration promotes *Veillonella’s* growth on organic acids and regulates its metabolic pool, enabling it to use amino acids and peptides as carbon sources. The growth of *Veillonella* is dependent on nitrate during intestinal inflammation, which is the primary factor determining the ability of extrenteral microorganisms to colonize the intestine. *Veillonella* utilizes the respiratory action of nitrate to ectopic colonize the intestine. *Veillonella* may promote colorectal cancer by aggregating inflammation in the gut (Rojas-Tapias et al., 2022). There was a study that showed the abundance of *Veillonella* was significantly increased in colorectal cancer by terminal restriction fragment length polymorphism and next-generation sequencing analysis and may act as an opportunistic pathogen and/or a driver of inflammation (Kasai et al., 2016). In addition, a metagenomic analysis of the gut microbiome based on an Indian population showed an abundance of *Veillonella* in colorectal cancer patients, significantly different from the normal population. Genetic and epigenetic changes in cancer may result from genotoxic stress to the gut microbiome or metabolites in the gut environment (Bamola et al., 2022). This contradicts our findings of *Veillonella* in healthy people, and further studies are needed to confirm the role of *Veillonella* in the gut.

Colorectal cancer occurs mostly through the adenomato-cancer pathway, but colorectal polyps do not have typical clinical features in the early stage of the disease. Therefore, early screening of polyps plays a key role in the prevention of colorectal cancer. The correlation between polyps and microbial characteristics can provide new ideas for early diagnosis. The occurrence and development of colorectal adenoma are closely related to the increase of *Prevotella* abundance. Increased *Prevotella* abundance is associated with increased T-assisted type 17 (Th17) mediated mucosal inflammation, promoting mucosal T immune response and neutrophil recruitment. Moreover, *Prevotella* can mediate mucosal inflammation leading to the systemic spread of inflammatory mediators, bacteria and bacterial products. In turn, it can exhibit more inflammatory properties, which are involved in the occurrence and development of diseases in the body (Larsen, 2017; Iljazovic et al., 2021). The influence of the gut microbiota on health and disease regulation is primarily through their metabolites. *Prevotella* possesses the enzymes and gene clusters necessary for the fermentation and utilization of complex polysaccharides and can produce major dominant metabolites such as acetate and propionic acid by fermentation (Di Pierro, 2021). *Prevotella* can efficiently hydrolyze plant fiber and has a low-fat, low-protein fermentation potential. *Prevotella* was highly sensitive to bile salts and highly dependent on bicarbonate. The main metabolic pathway of *Prevotella* is based on fumarate glycolysis and succinate production. Pyruvate can be degraded to acetate and formate (Franke and Deppenmeier, 2018). The diversity of *Prevotella* species is related to diet, lifestyle and geography (Tett et al., 2021). In our study, the abundance of *Prevotella* in the adenoma group was significantly increased compared with the control group by LEfSe analysis (Figure 3). Clinically, the occurrence of colorectal polyps and adenomas is closely related to dietary habits. Colorectal adenomas occur in patients with relatively control diets or vegetarians, and these patients have a reduced risk of colorectal adenomas (Guo et al., 2021; Nguyen et al., 2021). Studies have indicated that enterotypes is related to long-term diet, and the intestinal microbes that cause colorectal adenomas may be different among different enterotypes (Wu et al., 2011). Therefore, we speculate that the occurrence of colorectal polyps and adenomas is related to the promotion of inflammation and the change of long-term dietary habits.

Our study found that *Prevotella* enterotype was positively correlated with the occurrence of adenoma. This suggests that fecal flora is a potentially beneficial tool for colorectal cancer detection. Our study suggests that *Prevotella* may increase the incidence of colorectal adenoma, and that people classified as enteric *Prevotella* have a greater risk of adenoma. We provide a risk prediction model for colorectal adenoma based on *Prevotella* enterotype. Enterotypes differentiation may be helpful for precision medicine. There are some limitations to our study. First of all, the small sample size of the analysis means that the representativeness of our study is not ideal. Second, we only used one method to cluster fecal samples, and we did not identify the microbiome structure of other populations. Third, pathological and genetic heterogeneity of gut microbiome among different enrolled participants may be confounding factors in our study. Fourthly, our study lacks the validation of external cohort samples, which makes our study have certain limitations. In conclusion, the exact effect of enterotypes on colorectal adenoma needs to be further verified by large prospective studies and also confirmed in animal models. Nevertheless, our results provide a new direction for exploring the relationship between gut flora and colorectal adenoma.

## 5. Conclusion

For coastal people, *Prevotella* enterotype has a high risk of adenoma in high-risk population of colorectal cancer. *Prevotella* enterotype prompts people to make early diagnosis of adenoma, so as to warn such people to pay attention to the occurrence of adenoma, early diagnosis and treatment, and prevent the evolution of colorectal cancer. Further research is needed to determine whether *Vellonella* enterotype has a protective effect on the human gastrointestinal tract.

## Data availability statement

The datasets presented in this study can be found in online repositories. The names of the repository/repositories and accession number(s) can be found at: BioProject, PRJNA911829.

## Ethics statement

The studies involving human participants were reviewed and approved by the study was approved by institutional ethics board of the Sixth Affiliated Hospital, Sun Yat-sen University (NO. 2022ZSLYEC-121). The patients/participants provided their written informed consent to participate in this study.

## Author contributions

XG and JL study conception and design. JH, QZ, and DL administrative support. TX and XB acquisition of data. MS, ML, JZ, and JD analysis and interpretation of data. All authors contributed to the article and approved the submitted version.

## Funding

This work was supported by National Key Clinical Discipline, Natural Science Foundation of Tibet Autonomous Region, China (2031021016). Data analysis service were provided by Wekemo Tech Group Co, Ltd. Shenzhen China.

## Conflict of interest

The authors declare that the research was conducted in the absence of any commercial or financial relationships that could be construed as a potential conflict of interest.

## Publisher’s note

All claims expressed in this article are solely those of the authors and do not necessarily represent those of their affiliated organizations, or those of the publisher, the editors and the reviewers. Any product that may be evaluated in this article, or claim that may be made by its manufacturer, is not guaranteed or endorsed by the publisher.
